# [Retracted] Silencing of decoy receptor 3 (DcR3) expression by siRNA in pancreatic carcinoma cells induces Fas ligand-mediated apoptosis *in vitro* and *in vivo*

**DOI:** 10.3892/ijmm.2026.5971

**Published:** 2026-08-31

**Authors:** Jian Zhou, Shiduo Song, Songbin He, Zhenxin Wang, Bing Zhang, Dechun Li, Dongming Zhu

Int J Mol Med 32: 653-660, 2013; DOI: 10.3892/ijmm.2013.1437

Following the publication of the above article, a concerned reader drew the Editor's attention to the fact that, regarding the EdU assay experiments shown in Fig. 6 on p. 657, discrete areas of the cells appeared to overlap in different data panels, which were intended to show differently performed experiments in two distinct cell lines (the SW1990 and Panc-1 cell lines), albeit the areas were inverted in the case of the 'LV-RNAi' data panels. Moreover, within these areas of overlapping cells, certain of the cells were uniquely found to be coloured differently in the associated data panels. In addition, in Fig. 9 on p. 658, the bands shown for the caspase-3 and caspase-3 experiments with the SW1990 cell line looked remarkably similar, suggesting that the data in this figure may also have been assembled incorrectly.

After having conducted an internal investigation of the data in this paper, the Editor of *International Journal of Molecular Medicine* has decided that this article should be retracted from the publication on the grounds of an overall lack of confidence in the presented data. The authors were asked for an explanation to account for these concerns, but the Editorial Office did not receive a reply. The Editor sincerely apologizes to the readership for any incovenience caused, and we thank the reader for drawing this matter to our attention.

